# De-novo Antineutrophil Cytoplasmic Antibody-Associated Vasculitis Following the mRNA-1273 (Moderna) Vaccine for COVID-19

**DOI:** 10.7759/cureus.19616

**Published:** 2021-11-16

**Authors:** Elio Junior Feghali, Maha Zafar, Sidrah Abid, Dominick Santoriello, Swati Mehta

**Affiliations:** 1 Internal Medicine, Lebanese American University School of Medicine, Beirut, LBN; 2 Internal Medicine, Mayo Hospital, Lahore, PAK; 3 Nephrology, Albany Medical Center, Albany, USA; 4 Pathology & Cell Biology, NewYork-Presbyterian/Columbia University Irving Medical Center, New York, USA

**Keywords:** acute kidney injury, glomerulonephritis, mrna-1273 vaccine, coronavirus disease (covid-19), pr3-anca

## Abstract

Antineutrophil cytoplasmic antibody (ANCA) is a systemic autoimmune disorder characterized by antibodies directed against small- and moderate-sized vessels. While there are few reported cases of autoimmune illnesses associated with influenza vaccination, two cases of de-novo anti-proteinase (PR3) ANCA-associated pauci immune glomerulonephritis are reported after the mRNA-1273 coronavirus disease 2019 (COVID-19) vaccine. Here, we report the third case of ANCA-associated glomerulonephritis after the mRNA-1273 COVID-19 vaccine. Our patient presented with acute kidney injury and sub-nephrotic proteinuria four days after receiving the second dose of the COVID vaccine. He was found to have elevated c-ANCA and anti-PR3 antibodies. Renal biopsy confirmed focal necrotizing and diffuse crescentic glomerulonephritis. He was diagnosed with pauci immune glomerulonephritis. The patient achieved remission 10 weeks after the diagnosis with successful treatment.

## Introduction

The US Food and Drug Administration (FDA) has issued an emergency use authorization for three vaccines: Pfizer-BioNTech, Moderna, and Janssen/Johnson & Johnson for the coronavirus infectious disease 2019 (COVID 19).

The current data available from several large clinical trials indicate that the approved COVID-19 vaccines are safe and effective with mild local side effects along with few constitutional systemic side effects. Severe adverse effects are rarely reported [1-3].

There are few reports of ANCA-associated vasculitis following COVID-19 vaccination [4-9]. However, only two cases of de-novo anti-proteinase (PR3) ANCA-associated pauci immune glomerulonephritis are reported after the mRNA-1273 (Moderna) COVID-19 vaccine to date [8,9]. We hereby report a third rare case of ANCA-associated glomerulonephritis after the mRNA-1273 (Moderna) COVID-19 vaccine.

## Case presentation

A 58-year-old Caucasian (American) male with an unremarkable past medical history presented for evaluation of nausea, vomiting, and a 30-pound weight loss over the past two months at our hospital. He also reported dark-colored urine and intermittent episodes of hemoptysis during the same period. Specifically, he stated that his symptoms started four days after receiving his second dose of the mRNA-1273 (Moderna) vaccine for COVID-19. His first dose taken three weeks earlier was well tolerated. He denied any flank or abdominal pain, melena, fever, cough, hematuria, urinary frequency or urgency, and trauma. He denied smoking. Vital signs were stable upon admission. Physical examination was insignificant for any lower extremity pitting edema, petechiae, or rash. The patient was not on any medication prior to his hospitalization.

Laboratory analysis was remarkable for serum creatinine of 4.1 mg/dL (0.8-1.4 mg/dL) along with hematuria and sub-nephrotic proteinuria of 1796 g/24 hours (<150 mg/24 hours). Our differential diagnosis at this point was wide including all nephritic syndromes given AKI, hematuria and proteinuria. All serological workup was subsequently sent. C-ANCA (anti-neutrophil cytoplasmic antibodies) were elevated 160 AU/mL (20-25 AU/mL) and anti-proteinase 3 (anti-PR3) antibodies were also elevated >100 EU/ mL (normal <3.5 EU/mL) (Table 1). Immunohistochemical staining for the SARS-CoV-2 spike protein was not performed. All previous routine laboratory parameters including urinalysis were within normal range.

**Table 1:** Laboratory and serologic parameters on admission. WBC: white blood cells; BUN: blood urea nitrogen; HIV: human immunodeficiency virus; HBsAg: hepatitis B surface antigen; HCV: hepatitis C virus; ds DNA: double-stranded DNA; ANCA, antineutrophil cytoplasmic antibody; MPO: myeloperoxidase; PR3: proteinase 3.

Laboratory/serology parameter	Value	Reference range
WBC, 10^3^/ul	7,700	3,400-10,800
Hemoglobin, g/dl	9.8	13.6-16.7
Platelets, 10^3^/ul	385,000	130,000-350,000
Albumin, g/dl	2.5	3.5-5.2
Calcium, mg/dl	8.3	8.6-10.3
Phosphorus, mg/dl	4.1	2.4-4.7
Creatinine, mg/dl	3.98	0.8-1.4
BUN, mg/dl	51	7-22
Sodium, meq/l	141	135-145
Potassium, meq/l	3.8	3.4-5.2
Urine RBCs, per/hpf	20-50	0-2
Urine protein	2+	Negative
24-hour urine protein, grams/day	1796	<150
HIV ag/ab –titer	Negative	Negative
HBsAg-titer	Negative	Negative
Anti HCV-titer	Negative	Negative
Complement c3, mg/dl	90	87-200
Complement c4, mg/dl	25	19-52
Anti-DS DNA, Iu/ml	<12	<30
C ANCA - titer	160	<20
P ANCA - titer	<20	<20
Anti MPO, u/ml	<1.2	0-9.0
Anti PR3, u/ml	>100	0-3.5
Anti-glomerular antibody	4	>21
Kappa/lambda ratio	1.87	0.65-1.5

He underwent computed tomography (CT) scan of the chest for evaluation of hemoptysis that showed a right upper lobe consolidation and moderate bilateral pleural effusion.

**Figure 1:**
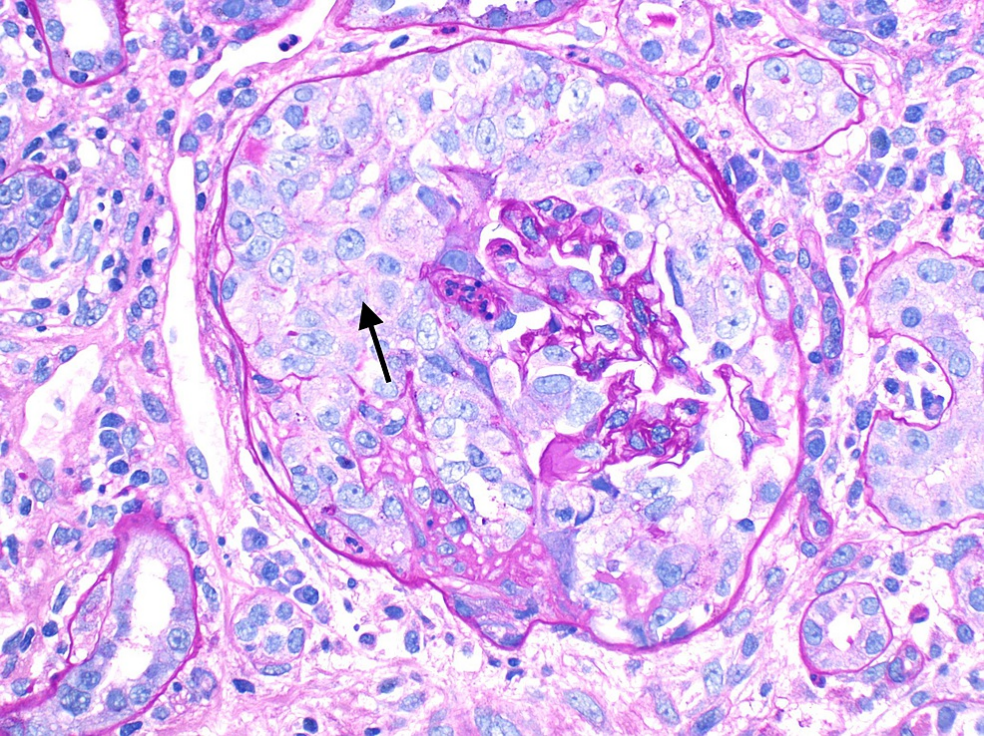
A glomerulus showing cellular crescent formation (Periodic acid Schiff, original magnification x 200).

**Figure 2:**
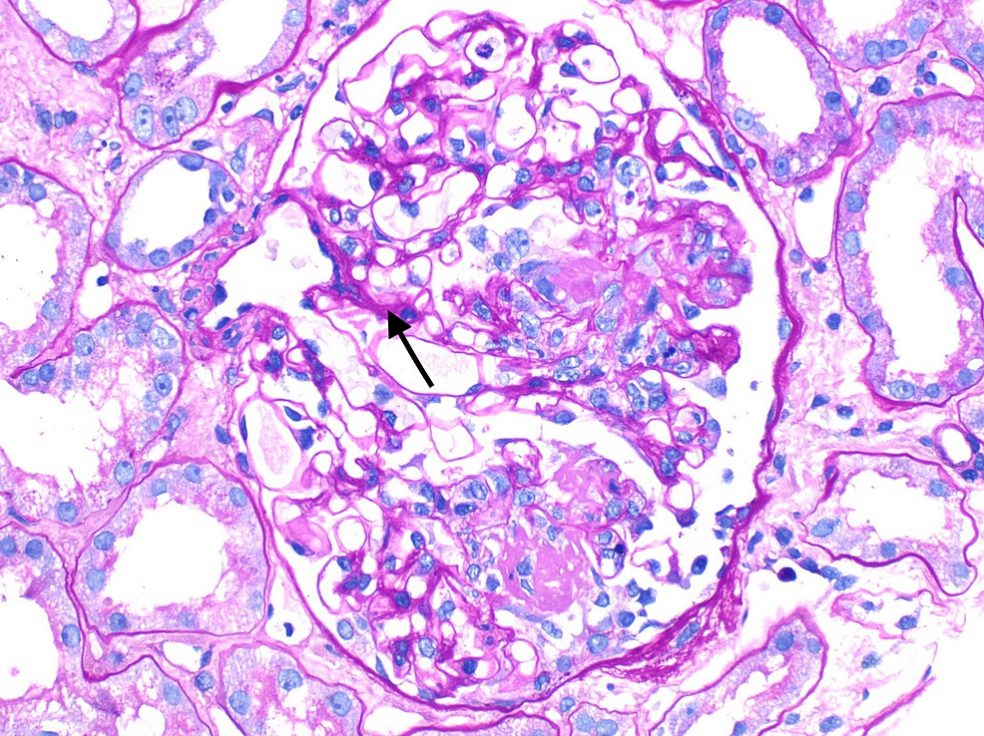
A glomerulus showing segmental fibrinoid necrosis of the tuft (Periodic acid Schiff, original magnification x 200).

The renal ultrasound was unremarkable. Renal biopsy was subsequently performed and showed acute, pauci immune, focal necrotizing, and diffuse crescentic glomerulonephritis (Figures 1, 2).

The patient was diagnosed with anti-PR3-associated ANCA glomerulonephritis. He received intense immunosuppression with plasma exchange x 5 cycles, intravenous prednisone 1 gram x 3 doses followed by prednisone 60 mg daily, cyclophosphamide x 2 doses, and rituximab x 4 doses. The patient achieved remission after 10 weeks of diagnosis with a resolution of symptoms and improvement in renal function with a creatinine of 1.5 mg/dl. He is following in our nephrology office regularly since discharge. 

## Discussion

ANCAs have been implicated in the pathogenesis of ANCA-associated vasculitis (AAVs) and are present in 90% of the cases [10]. However, the etiology and pathogenesis leading to the initiation of the AAVs remain poorly understood. Several environmental, infectious, medicines, genetic factors have been implicated in the pathogenesis of AAV [11].

Influenza vaccine has been associated with autoimmune illnesses including leukocytoclastic vasculitis, Henoch-Schönlein purpura, giant cell arteritis and AAV in predisposed individuals with pre-existing autoimmune diseases [12]. Transient systemic inflammatory cytokine response, molecular mimicry, and polyclonal activation are all possible mechanisms that could theoretically explain this temporal relationship [13].

Recently, a few cases of AAV following COVID-19 vaccination are being reported in the literature including ANCA-associated glomerulonephritis and AAV following Pfizer-BioNTech [4-6], AAV following Oxford-Astrazeneca vaccine [7], ANCA glomerulonephritis and AAV following Moderna vaccine [8,9]. The latter two cases both presented with AKI, sub-nephrotic proteinuria and elevated anti-proteinase 3 (anti-PR3) antibodies. We hereby report another rare case of de-novo PR3-ANCA-associated pauci immune glomerulonephritis following the mRNA-1273 (Moderna) vaccine for COVID-19 to further extend our knowledge and understanding of this rare association.

Although the exact pathophysiology remains obscure, it is possible that the enhanced immune response observed after the second dose of COVID-19 vaccination could be responsible for triggering the ANCA ultimately leading to AAV. Molecular mimicry and cross-reactivity have been considered as a trigger for autoinflammatory diseases [14]. COVID-19 patients were found to be at a higher risk of developing autoimmune diseases including systemic lupus erythematosus, Guillain-Barre syndrome, and Kawasaki disease [15]. Several cases of ANCA-associated glomerulonephritis and AAV occurring in patients with COVID-19 have been described [16,17], supporting the hypothesis that the virus itself, or the immune response to it, could trigger the development of ANCA, leading to AAV.

Although our case does not prove causality and it is possible this association is coincidental given millions of people around the world are now vaccinated, the fact that our patient did not have any symptoms prior to the vaccination along with normal lab values raises a high index of suspicion for the observed correlation. Nevertheless, AAV in association with COVID-19 and vaccination have been reported; therefore, further investigation is required to understand the underlying mechanism linking the COVID-19 vaccine and AAV.

## Conclusions

We report an extremely rare case of anti-PR3-associated ANCA glomerulonephritis after mRNA-1273 (Moderna) COVID-19 vaccination. There is a need for increased awareness amongst clinicians to recognize this rare disease in patients presenting with renal failure after vaccination in the right clinical context. Further research is required to understand the underlying mechanisms linking the COVID-19 vaccine to AAV if any.
